# Carbon Nanostructures for Ocular Tissue Reinforcement

**DOI:** 10.1167/tvst.11.9.1

**Published:** 2022-09-01

**Authors:** Joaquin Silvestre, Shihao Chen, Zheng Zheng, Alfredo Vega, Tong Chen, Francisco Rodríguez-Reinoso, Pin Zhu, Shuang Zeng, Yaru Zheng, Fangjun Bao, Yong Liu, Jorge L. Alió

**Affiliations:** 1 Laboratorio de Materiales Avanzados, Departamento de Química Inorgánica-IUMA, Universidad de Alicante, Spain; 2 Eye Hospital, Wenzhou Medical University, Wenzhou, Zhejiang, China; 3 Research and Development Department, VISSUM Corp., Alicante, Spain

**Keywords:** carbon nanostructures, keratoconus, cornea, ocular tissue reinforcement, nanotechnology

## Abstract

**Purpose:**

The purpose of this study was to improve the biomechanical properties of the cornea through the incorporation of carbon nanostructures.

**Methods:**

Healthy Japanese rabbits were used to evaluate the effect of carbon nanostructures’ incorporation in the cornea. Rabbits were divided in two groups A and B. In each of these groups, the corneas were divided in (i) corneas not submitted to any treatment (the control group), (ii) corneas modified either with carbon nanostructures (group A), or with the traditional cross-linking technology (group B). After modification, rabbits were euthanized at different time intervals. The biomechanical properties of the treated corneas were evaluated using the inflation method.

**Results:**

Biomechanical tests based on the inflation method show that the incorporation of carbon nanostructures to the cornea and their proper distribution within it gives rise to a large improvement in the mechanical properties and tangential elastic modulus (up to 155%). These results anticipate that this novel and easy approach based on nanotechnology is able to compete with the actual cross-linking technology applied in clinical ophthalmology using a photosensitive molecule, such as riboflavin and unpleasant UV-A radiation.

**Conclusions:**

The incorporation of carbon nanostructures (single-walled carbon nanotubes and graphene) in corneal stroma is proposed as a promising alternative to improve the mechanical properties in the treated eyes. The proper dispersion of the carbon nanostructures a few days after implementation (down to 60 micrometers depth) explains the successful results achieved.

**Translational Relevance:**

Nanotechnology applied to the eye constitutes a promising approach for ocular tissue reinforcement.

## Introduction

Keratoconus is a disease associated with a severe thinning and deformation of the cornea, thus resulting in blurry vision, double vision, astigmatism, and light sensitivity. Currently available treatments based on rigid contact lens, lamellar Keratoplasty, and intacs involve surgery and do not treat the underlying cause of ectasia. A novel approach introduced in the last few years is corneal collagen cross-linking (CXL). This approach is based on the application of a photosensitizer, such as riboflavin and ultraviolet irradiation (UV-A) light (370 nm).^1^ The main purpose of this treatment is to increase the cross-linking within and between collagen fibers using photopolymerization, with the associated improvement in the mechanical properties of the cornea. Although cross-linking of corneal collagen is a promising approach for the treatment of keratoconus and secondary ectasia, several long-term and short-term complications have been documented, in addition to the patient inconvenience due to the use of UV-A irradiation.^2^ These complications include secondary infections, formation of temporary corneal haze, permanent scars, and endothelial damage, among others. In addition, UV-A irradiation can cause keratocyte and corneal endothelial cell destruction or death, together with lens and retinal damage. Considering the important incidence of keratoconus worldwide (around 50 to 230 cases per 100,000 population),^3^ the development of novel, noninvasive approaches, able to be applied without surgery or without inconvenient treatments will be a step-stone in ophthalmology.

A potential approach never evaluated in the literature concerns the use of nanomaterials and nanotechnology in the field of ophthalmology.^4^^–^^6^ Indeed, the incorporation of exogenous nanodevices or nanomaterials into living organisms constitutes a revolutionary methodology to treat diseases, disorders, failures, etc., thus opening the gate to a new field of research, the so-called nanomedicine.^7^ Actually, nanomedicine is mainly applied in the transport and delivery of target molecules (e.g. drugs) or biological agents, sensors, diagnosis and monitoring, and tissue engineering, among others.^8^

Among the potential materials to be applied in nanomedicine, carbon-based materials have been widely investigated with promising results in a number of applications.^9^^–^^11^ These include mainly graphene and carbon nanotubes, applied as biosensors, molecular carriers, imaging devices, neuronal growth platforms, etc.^12^^,^^13^ These carbon materials are characterized by exceptional properties, easily tailored by pre- and post-synthesis treatments, thus widening the versatility of these materials in nanomedicine (mainly modification in their surface chemistry through covalent and noncovalent anchoring of biological molecules). For instance, graphene is considered an extremely hard material (it is about 200 times higher than steel); it has a proper elasticity, minimizing the risk of breakage; it is almost transparent; it has excellent thermal and electrical properties; and it has antibacterial properties, among others. Carbon nanotubes (CNTs) are also emerging nanomaterials with great applicability in biomedicine and biotechnology due to the unique characteristics of these one-dimensional materials (hollow materials with a high surface area, excellent thermal and electrical properties, high surface/volume aspect ratio, high tensile strength, etc.). Depending on the number of rolled-up graphene sheets, CNTs can be classified as single- (SWCNTs), double- (DWCNTs), or multiwalled (MWCNTs) nanotubes. These structural characteristics are highly important because they will define the drug-loading capacity, optical and mechanical properties (CNTs are at least 100 times as strong as steel), transparency, and toxicity (SWCNTs have in general more outstanding properties).^14^^–^^16^ The integration of SWCNTs into thin polymeric films has been proposed as a promising route to achieve unique electrical and mechanical properties, while preserving optical transparency.^17^^–^^19^ The incorporation of MWCNTs into polycaprolactone (PCL) allowed to increase the tensile and compressive strength of the composite scaffolds, with high potential for bone tissue regeneration.^20^ CNTs have also been applied to reinforce polymethyl methacrylate (PMMA) bone cement to be used in orthopedic surgery.^21^ Incorporation of nanotubes improve the fatigue performance of the PMMA bone cement and encourage interfacial cell growth. Mikael et al. have shown that the incorporation of MWCNTs into poly(lactide-co-glycolide) (PLGA) polymer scaffolds can improve the compressive strength and modulus more than 100% and 200%, respectively, compared to pure PLGA scaffolds.^22^

Despite these excellent results described so far in the application of carbon nanomaterials in nanomedicine, to our knowledge, these systems have never been applied in ophthalmology. Previous studies dealing with artificial collagen-carbon nanotube composite materials have shown that these matrices can be very useful scaffolds in tissue engineering, or as components in biosensors or other medical devices.^23^ SEM images of the collagen-SWCNT composite matrix showed the presence of proper physical interactions between the two components, without changes in cell viability or cell proliferation. Based on these results and considering that the eye is mainly composed of collagen fibers, it is possible to anticipate that carbon nanostructures could have a potential impact in a number of ocular disorders, from drug delivery applications to ocular tissue reinforcement, provided that they can be properly incorporated and distributed within the eye. Preliminary studies from our research group demonstrated that the incorporation and distribution of the carbon nanostructures in the cornea is not easy.^24^ In these initial stages, the nanostructures were incorporated using unpleasant dissection of the corneal stroma to create a pocket at 300 microns in depth.

Based on these preliminary studies, the aim of the present study is to evaluate the biomechanical properties of corneas before and after being treated with carbon nanomaterials avoiding undesirable dissections, and to evaluate the accessibility and distribution of these nanostructures within the cornea when incorporated topically. To this end, a combination of SWCNTs and graphene will be used. The main goal of our approach is to take advantage of both materials once incorporated in the cornea (i.e. flexibility and antibacterial properties of graphene and penetration ability and transparency of SWCNTs).

## Materials and Methods

### Carbon Nanostructures

Carbon nanostructures applied in this study include SWCNTs and graphene. Purified SWCNTs (97% carbon) were purchased from Nano-C (Westwood, MA) and graphene (highly reduced graphene oxide; oxygen content approximately 1.5%) was purchased from Avanzare S.L. (Spain). Carbon nanomaterials were used as received without any additional purification treatment.

A suspension of the carbon nanostructures (CNS) was prepared by weighting 2 mg of both carbon components (50:50) in a sterilized glass container and adding 7 mL of physiological solution BSS. Although initially the carbon nanostructures tend to aggregate due to their hydrophobic character, a sonication treatment for 10 minutes was applied to achieve a proper dispersion. Once dispersed, the suspension was stored in the refrigerator until use.

### Characterization Methods

Transmission electron microscopy (TEM) analysis of the received samples were performed in a JEOL microscope JEM-2010 at the University of Alicante. Raman analysis was performed using an NRS-5100 spectrometer (Jasco) equipped with a laser radiation source of 532 nm and a CCD detector.

The incorporation of the nanostructures in the cornea was performed using healthy Japanese Rabbits from the Animal Breeding Unit at Wenzhou Medical University. Initially, the epithelium was removed by a scraper. Rabbits were divided in two groups (group A [*n* = 25] and group B [*n* = 15]). In group A, additional punctures (15 punctures of around 100 µm in depth) were performed in the cornea using a 30-gauge needle to promote the penetration of the nanostructures, without compromising the mechanical properties of the cornea. In group A, one of the eyes was infiltrated with the carbon nanomaterials suspension (0.28 mg/mL; A-CNS), while the other one was infiltrated only with PBS solution (A-SS). In group B, one of the eyes was submitted to the traditional cross-linking technology using riboflavin (5.4 J/cm^2^; 0.22% concentration by volume) and UV-A (370 nm, 3 mW/cm^2^) for 30 minutes (B-3mW), while the other eye was not exposed to any treatment (neither riboflavin nor irradiation [B-blank]). After de-epithelialization, and puncture wounds of 100 microns, the eyes were infiltrated with the carbon nanomaterials suspension or phosphate-buffered saline (PBS) solution for comparison. After infiltration, the eyes were washed with PBS. Subsequently, Tobramycin Eye Drops (three times daily [t.i.d.], 7 days), Deproteinized Calfblood Extract Eye Gel (t.i.d., 7 days), and Tobramycin Dexamethasone Eye Gel (every night [qn], 3 days) were applied into the treated eyes. The rabbits were then fed normally for different days (e.g. 1 day, 3 days, 2 weeks, and 3 months) until rabbits are euthanized by air embolism. The eyeballs were then removed at once. The corneoscleral tissue along the limbus was cut and fixed in the anterior chamber simulator for study. Table 1 contains a summary of the different groups evaluated.

**Table 1.** Summary of Experiments Performed in the Evaluated Rabbits

Group	Treatment	*n*
A-SS	PBS, 1 min	25
A-CNS	CNS solution, 1 min	25
B-blank	No treatment	15
B-3mW	Riboflavin + UV-A 30 min	15

The distribution of carbon nanostructures within the cornea was evaluated using TEM and Raman. TEM analyses were performed in a Hitachi H7500 microscope at the Wenzhou Medical University and Raman analysis were performed using a Nicolet Almega equipment (Thermal Scientific) located at the Nanotechnology Laboratory of Wenzhou Medical University.

The mechanical properties of the modified corneas were evaluated using biomechanical inflation testing. To this end, the corneas were mounted onto a custom-built pressure chamber used for corneal inflation testing. The pressure chamber was filled with phosphate buffered saline solution (Maixin, China) and connected to a syringe pump, which in turn was connected to a motor whose movement was controlled by bespoke LabView software. The pressure was controlled by the movement of the motor and continuously monitored using a pressure transducer (DMP-HS, Hangzhou, China) that connected with the pressure chamber. Side images of the corneal profile were recorded with digital cameras (EOS 60D, Canon, Inc., Tokyo, Japan) positioned in the three directions (120 degrees apart). The initial profiles and values of corneal diameters and thicknesses measured at 2 mm Hg pressure were used to construct a numerical model of each corneal specimen. To ensure a fully inflated and wrinkle-free corneal surface, all specimens were subjected to an initial inflation pressure of 2 mm Hg. Connected to a personal computer to record the data automatically, a charge-coupled device laser displacement sensor (LK series; Keyence, Itasca, IL) was used to monitor the displacement at the corneal apex continually. Each specimen was tested within 3 hours postmortem. To condition and stabilize the behavior, 3 cycles of loading and unloading up to a pressure of 50 mm Hg were applied at a rate of 0.4 mm Hg/sec. A recovery period of 90 seconds was allowed between each of the 2 loading cycles to ensure the behavior was not affected by the strain history of loading cycles. Finally, the specimens were subjected to a fourth loading cycle (0.4 mm Hg/sec, maximum 50 mm Hg), the results of which were used in a subsequent inverse analysis.

An inverse analysis process was used to evaluate the material's mechanical properties of corneal tissue based on the pressure-deformation experimental results. As described in a previous study,^25^ the finite element solver Abaqus (Dassault Systèmes Simulia Corporation, Forest Hill, MD) and optimization software package LS-OPT (Livermore Software Technology Corporation, Livermore, CA) were used to implement the iterative process of the inverse analysis procedure. Eighty-four finite element, specimen-specific models, each using 1728, fifteen-noded continuum elements (C3D15H), arranged in 12 rings and 2 layers, were developed from the specimens’ initial geometry based on their thickness, corneal profile, and diameter measurements. An encastre connection was assumed along the limbus to simulate connection to the mechanical clamps. A first-order hyperelastic Ogden model was used to represent corneal material behavior using a strain energy function of the form:

(1) $$W\;=\;\frac{2\mu }{a^{2}}\;\left({\bar{\lambda }}_{1}^{\alpha }+{\bar{\lambda }}_{2}^{\alpha }+{\bar{\lambda }}_{3}^{\alpha }-3\right)+\frac{1}{D}{\left(J-1\right)}^{2}$$

where W represents the strain energy per unit volume, $\bar{\lambda_{k}}$ the deviatoric principal stretches = J^−1/3^*x* λ_*k*_ (k = 1, 2, 3), λ_1_, λ_2_, and λ_3_ the principal stretches, J = λ_1_λ_2_λ_3_. Material parameters µ and α are the shear modulus and the strain hardening exponent, respectively. D is a compressibility parameter = $\frac{3(1-2\nu )}{\mu \;(1+\nu )}$ calculated assuming corneal tissue was nearly incompressible with a Poisson's ratio, ν, of 0.48. Supplementary Figure S1 shows representative images of the inflation system used.

## Results

### Analysis of the Carbon Nanostructures

The morphology and the characteristics of the commercial carbon nanostructures was evaluated using TEM and Raman spectroscopy. The main purpose of the characterization is to confirm the purity and quality of the carbon nanostructures and to gain more knowledge about their morphological characteristics. Supplementary Figure S2 shows the TEM images for the SWCNTs and the graphene. Based on the specifications of Nano-C company, these carbon nanotubes (PT-100) have been purified to more than 95% carbon using acid leaching and oxidation, the remaining content being due to the residual iron catalyst. As it can be appreciated, SWCNTs are single tubes (only one carbon layer) with external diameter between 0.9 and 1.3 nm, and with a total length of around 1 µm. Even though samples are commercialized as purified SWCNTs, TEM images show that some residual catalyst is still present (<5%; i.e. iron nanoparticles can be clearly appreciated as black dots). Concerning graphene, the TEM image shows the presence of thin graphene sheets with dimensions around 0.5 µm × 1 µm. These images confirm that graphene exhibits a large purity (>99%, based on commercial specifications).

The characterization of the commercial samples has been completed with Raman analysis. The main purpose is to identify the quality of the carbon materials and the number of graphene layers. Supplementary Figure S3 shows the Raman spectra for the SWCNTs and graphene. SWCNT exhibits the traditional features described before in the literature with the main peak at 1590 cm^−1^ (G band), associated with vibrations of carbon atoms along the nanotube axis. Additionally, the main contribution has a shoulder at 1570 cm^−1^, associated with vibrations about the circumference of the nanotube. The presence of defects and sp^3^ hybridized carbon atoms give rise to the D band at 1360 cm^−1^, its small height confirming the large quality of the SWCNTs. Last but not least, the Raman spectrum shows a resonant 2D peak at 2600 cm^−1^ occurring as an overtone of the D band and the radial breathing modes (RBMs) at 200 to 300 cm^−1^. The spectrum for graphene shows the main characteristic bands at 1360 cm^−1^ and 1605 cm^−1^ attributed to the D and G-bands. In addition, the spectrum shows the 2D band at 2700 to 2900 cm^−1^. The broad nature of the 2D band and its low intensity clearly reflects that the commercial graphene is no single layer but rather contains several layers of graphene.

### Biomechanical Properties of the Modified Corneas

One of the main issues when evaluating the mechanical properties of modified corneas is to choose the proper biomechanical test able to mimic the real scenario taking place in human eyes suffering from keratoconus. The traditional stress-strain tests provide a measure of the deformation in the direction parallel to the eye surface upon application of a gradual load. In the specific case of corneas modified with interfibrillar agents (e.g. carbon nanostructures), the traditional biomechanical stress-strain tests will not be very useful because it will provide, in a last step, the mechanical properties of the individual collagen fibers themselves, this situation being far from the real scenario in the eye. With these premises, biomechanical inflation tests described in the experimental section are more realistic because these tests will measure the deformation of the cornea and the tangential elastic modulus upon application of a randomly distributed pressure, perpendicular to the eye curvature, in each point of the cornea. For the biomechanical tests, rabbits have been divided in four groups (see Table 1 in the Materials and Methods section).

**Figure 1.**
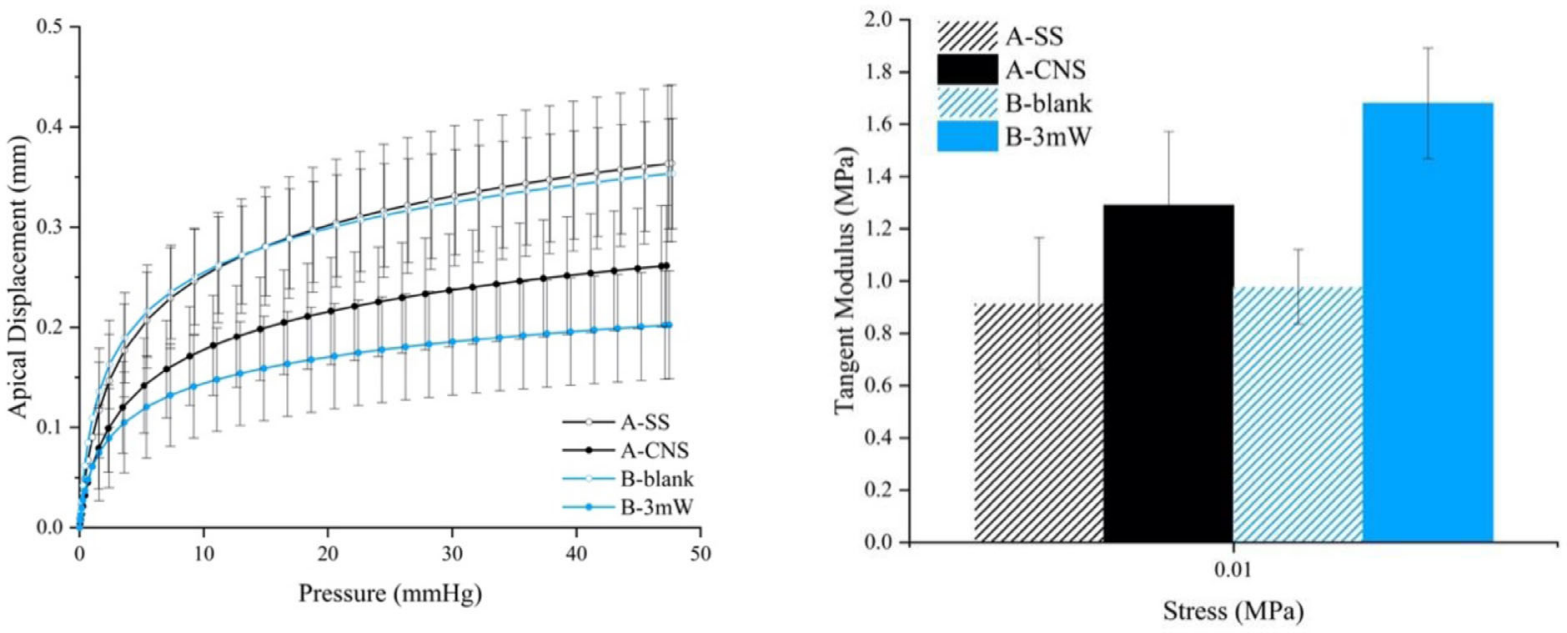
Biomechanical evaluation of the modified corneas (3 months after incorporation) submitted to the inflation tests. These tests include the control cornea (B-blank), the cornea treated with saline solution (A-SS), and the corneas modified with the traditional cross-linking technology (B-3mW) and modified with carbon nanostructures (A-CNS).

As it can be appreciated in Figure 1 and Table 2, the corneas used as a control (i.e. those without any treatment [B-blank] and those treated with saline solution [A-SS]), exhibit poor mechanical properties with a large deformation after inflation upon application of a pressure from 0.001 MPa up to 0.01 MPa. Interestingly, the scenario changes significantly after application of the traditional cross-linking technology. In this case (sample B-3mW), the improvement in the strength of the treated cornea (improvement in the tangent elastic modulus) is as high as 198% at 0.001 MPa and 172% at 0.01 MPa. The excellent performance after application of riboflavin under UV-light conditions has been widely described in the literature and at the moment constitutes the main technology applied in humans to treat keratoconus.

**Table 2.** Tangent Elastic Modulus at Different Stresses in Four Groups (MPa)

	Stress (MPa)									
Group	0.001	0.002	0.003	0.004	0.005	0.006	0.007	0.008	0.009	0.01
A-SS	0.1391 ± 0.0332	0.2192 ± 0.0496	0.3034 ± 0.0732	0.3892 ± 0.0984	0.4759 ± 0.1240	0.5631 ± 0.1498	0.6505 ± 0.1756	0.7381 ± 0.2014	0.8258 ± 0.2272	0.9136 ± 0.2530
A-CNS	0.2154 ± 0.0752	0.3260 ± 0.0710	0.4419 ± 0.0839	0.5605 ± 0.1067	0.6805 ± 0.1337	0.8015 ± 0.1625	0.9230 ± 0.1921	1.0449 ± 0.2221	1.1671 ± 0.2522	1.2895 ± 0.2825
Percentage 1 (%)										
	155	149	146	144	143	142	142	142	141	141
Difference 1	0.0763 ± 0.0791	0.1067 ± 0.0813	0.1385 ± 0.0935	0.1713 ± 0.1118	0.2046 ± 0.1333	0.2384 ± 0.1564	0.2724 ± 0.1805	0.3068 ± 0.2051	0.3413 ± 0.2300	0.3759 ± 0.2552
P1	<0.001^*^	<0.001^*^	<0.001^*^	<0.001^*^	<0.001^*^	<0.001^*^	<0.001^*^	<0.001^*^	<0.001^*^	<0.001^*^
B-blank	0.1313 ± 0.0363	0.2214 ± 0.0385	0.3143 ± 0.0476	0.4084 ± 0.0595	0.5030 ± 0.0726	0.5978 ± 0.0862	0.6928 ± 0.1001	0.7879 ± 0.1141	0.8829 ± 0.1282	0.9780 ± 0.1424
B-3mW	0.2595 ± 0.0934	0.4061 ± 0.0903	0.5598 ± 0.0960	0.7168 ± 0.1073	0.8756 ± 0.1218	1.0355 ± 0.1382	1.1960 ± 0.1557	1.3570 ± 0.1739	1.5183 ± 0.1926	1.6798 ± 0.2116
Percentage 2 (%)										
	198	183	178	176	174	173	173	172	172	172
Difference 2	0.1283 ± 0.1089	0.1847 ± 0.1024	0.2455 ± 0.1058	0.3084 ± 0.1151	0.3727 ± 0.1280	0.4377 ± 0.1430	0.5032 ± 0.1594	0.5691 ± 0.1767	0.6353 ± 0.1945	0.7017 ± 0.2128
P2	<0.001^*^	<0.001^*^	<0.001^*^	<0.001^*^	<0.001^*^	<0.001^*^	<0.001^*^	<0.001^*^	<0.001^*^	<0.001^*^
P3	0.09	0.011^*^	0.002^*^	0.001^*^	<0.001^*^	<0.001^*^	<0.001^*^	<0.001^*^	<0.001^*^	<0.001^*^

Percentage 1 (%) = (A-CNS)/(A-SS) difference 1 = (A-CNS) - (A-SS).

Percentage 2 (%) = (B-3mW)/(B-blank) difference 2 = (B-3mW) - (B-blank).

P1 value between differences of group A-SS and group A-CNS.

P2 value between differences of group B-blank and group B-3mW.

P3 value between differences of difference 1 and difference 2.

* Difference with statistical significance.

Figure 1 also shows the performance of the corneas treated with carbon nanostructure (A-CNS). Interestingly, our approach based on nanotechnology applied to cornea is also promising to improve the mechanical properties. As it can be appreciated, the cornea treated with carbon nanostructures exhibits a significant reduction in the corneal displacement after application of an inflation pressure. Compared to the control eyes, these results constitute a 155% and 141% improvement in the tangent elastic modulus at 0.001 and 0.01 MPa, respectively.

### Physicochemical Characterization of the Modified Corneas

**Figure 2.**
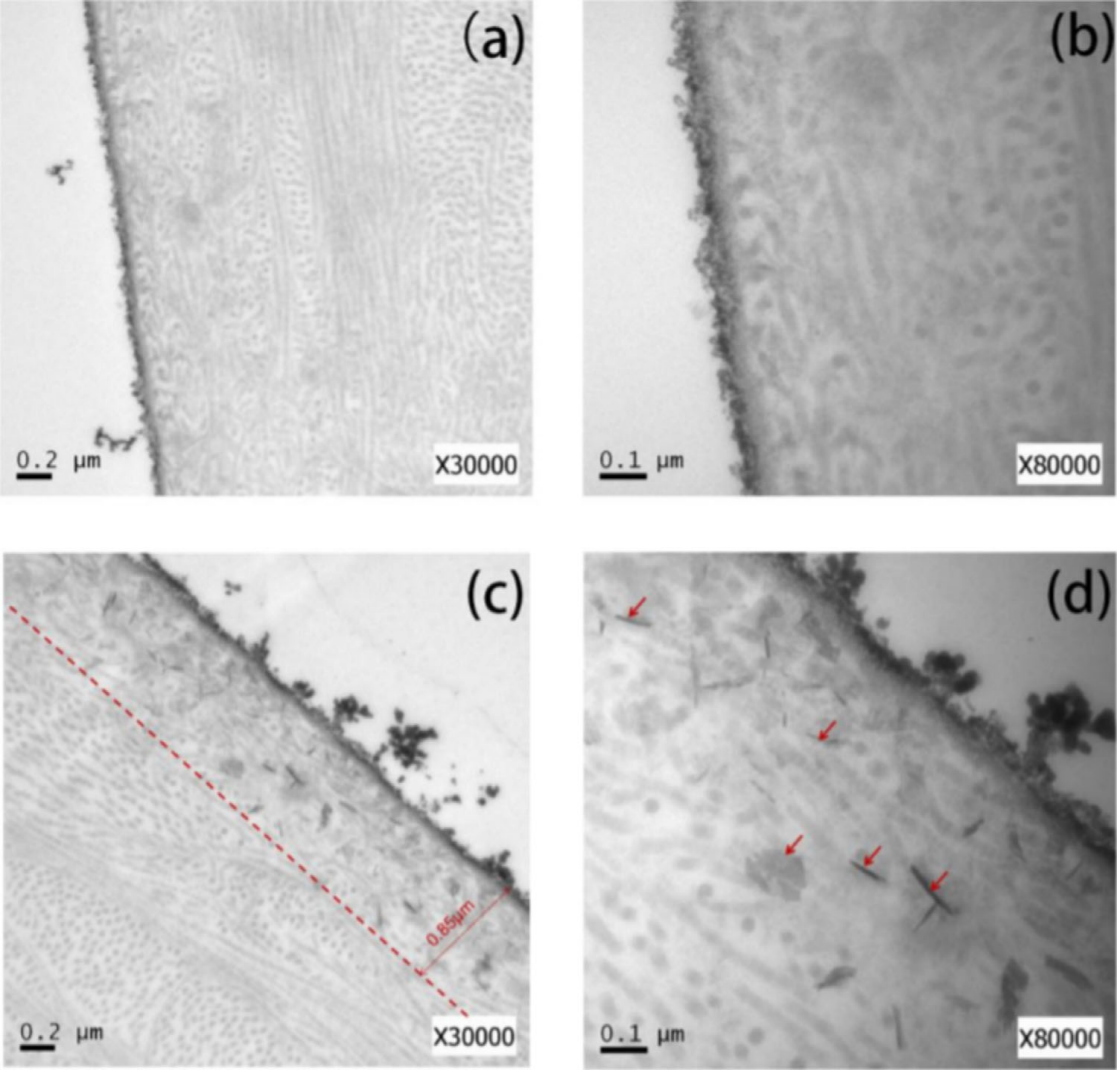
TEM images of the different corneas evaluated (**A, B**) control group and (**C,** **D**) corneas modified with carbon nanostructures (group A). *Red arrows* identify the location of the carbon nanostructures.

Once our approach has been validated, the open question at this point is to ascertain the reasons behind the improvement in the mechanical properties. One of the main concerns is the accessibility of the carbon nanostructures to the interior of the cornea and their distribution within it. To identify the presence of carbon nanostructures, two different techniques have been applied: TEM and Raman spectroscopy. The use of Raman is very useful to identify carbon nanostructures due to the presence of characteristic peaks for graphene and carbon nanotubes at around 1340 cm^−1^ (D band) and around 1572 cm^−1^ (G band), as described above. Figure 2 shows TEM images of the cross-section of the cornea 1 day postoperative.

**Figure 3.**
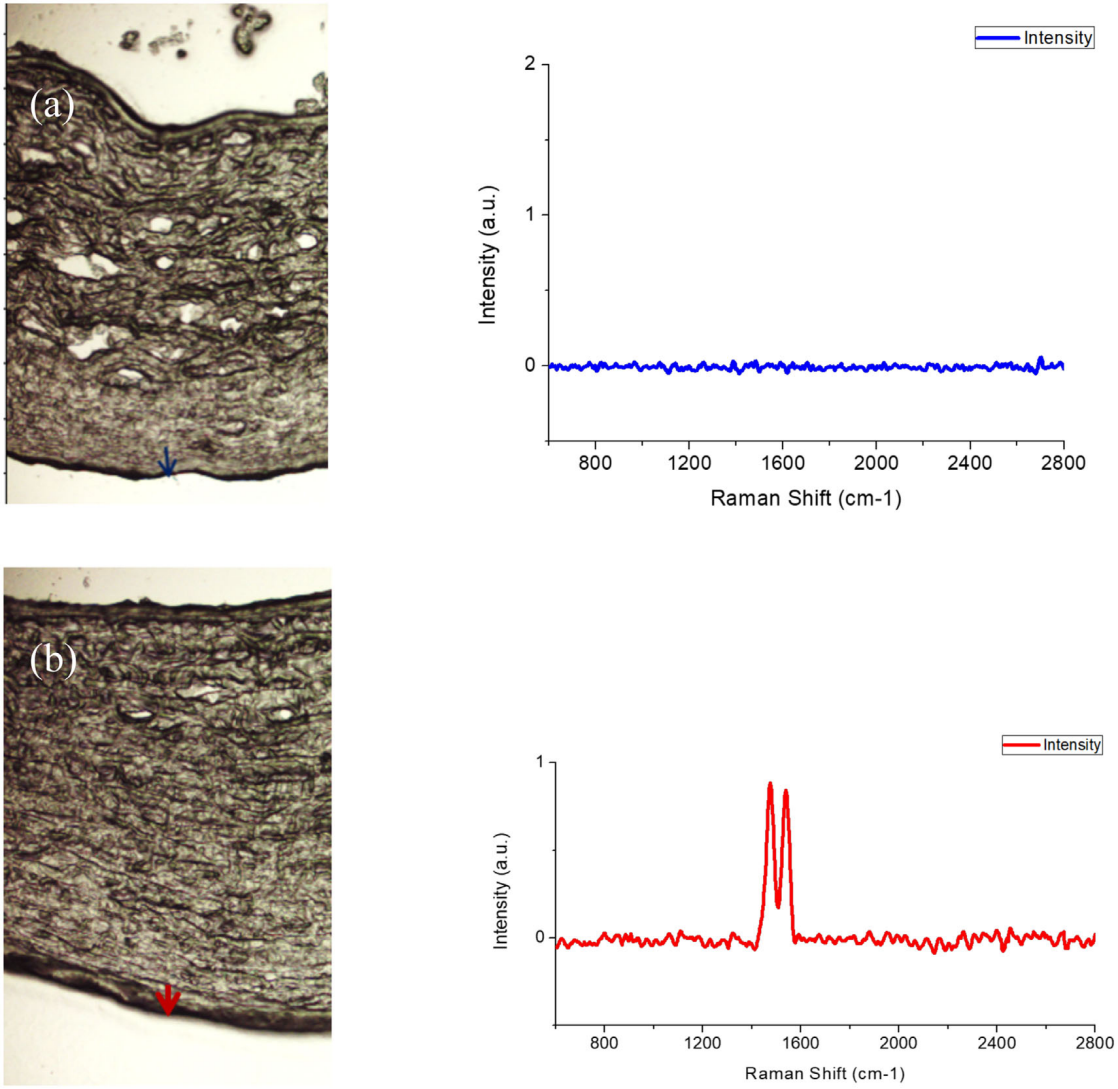
Raman spectra of (**A**) control group and (**B**) samples treated with carbon nanostructures (group A - 1 day after incorporation). *Red* and *blue arrows* identify the position for the Raman analysis.

As it can be observed in Figure 2, the control corneas (a and b) do not show any sign of carbon nanostructures. The analysis of the external surface section shows the fibrils due to the collagen but without presence of any external agent. In the case of the treated corneas, the scenario changes drastically. TEM images clearly show the presence of carbon nanostructures randomly distributed within the first 0.85 µm depth. The presence of carbon nanostructures, either in a rod-shape or as a spherical aggregate, can be clearly appreciated with red arrows. These results anticipate that even after 1 day, carbon nanostructures have been able to diffuse within the first section of the cornea (0.85 µm depth) with a proper distribution. To confirm that the observed fragments correspond to carbon materials and their nature, in situ Raman spectra have performed paying attention to the external layers (see arrows in Figure 3 to identify the location of the Raman analysis) of the treated and untreated corneas.

As it can be appreciated in Figure 3, the untreated cornea does not show any peak or signal active in the Raman spectra, the whole spectra being rather flat. On the contrary, the cornea modified with the carbon nanostructures clearly shows the main Raman peaks at 1590 cm^−1^ and 1570 cm^−1^, associated with the G band of the carbon nanostructures. The low intensity of the embedded nanostructures and the overlapping of the G band in graphene and SWCNTs does not allow to clearly identify the nature of the observed carbon nanostructures, although the doublet could anticipate some aggregation of graphene nanostructures in this specific section. This result will anticipate, a priori, a deeper penetration of SWCNTs in the cornea, whereas graphene could remain preferentially in the external layers.

To further confirm this point and identify the different diffusional regimes for the evaluated carbon nanostructures, similar experiments were performed for the treated corneas 3 days after incorporation of the nanostructures. Raman spectra were evaluated at different positions of the cornea A and B at a distance of 10 µm in depth.

**Figure 4.**
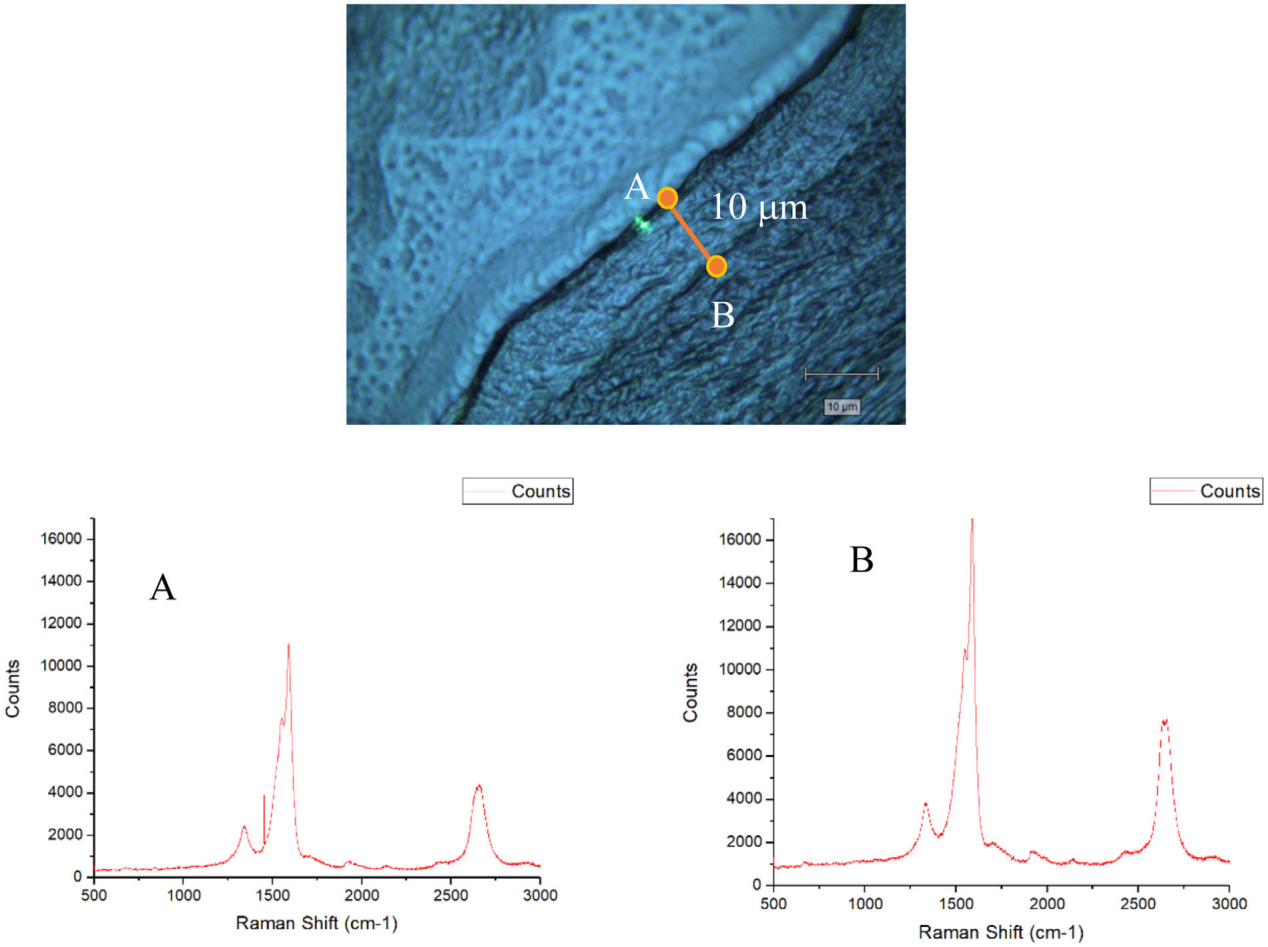
Raman spectra of samples treated with carbon nanostructures (group A - 3 days after incorporation) at positions A and B.

As it can be observed in Figure 4, three days after implementation it is possible to identify carbon nanostructures in the cornea stroma up to 10 µm from the surface (Position B, Fig. 4). The Raman spectra clearly shows that these nanostructures correspond mainly to SWCNTs, their concentration being larger at 10 µm from the surface (position B). These results clearly anticipate that the incorporation of the carbon nanostructures into the cornea is an activated process that improves with time, the penetration being highly promoted for nanotubes as compared to graphene. Last but not least, TEM analyses were performed in the treated and untreated corneas 2 weeks postoperative.

**Figure 5.**
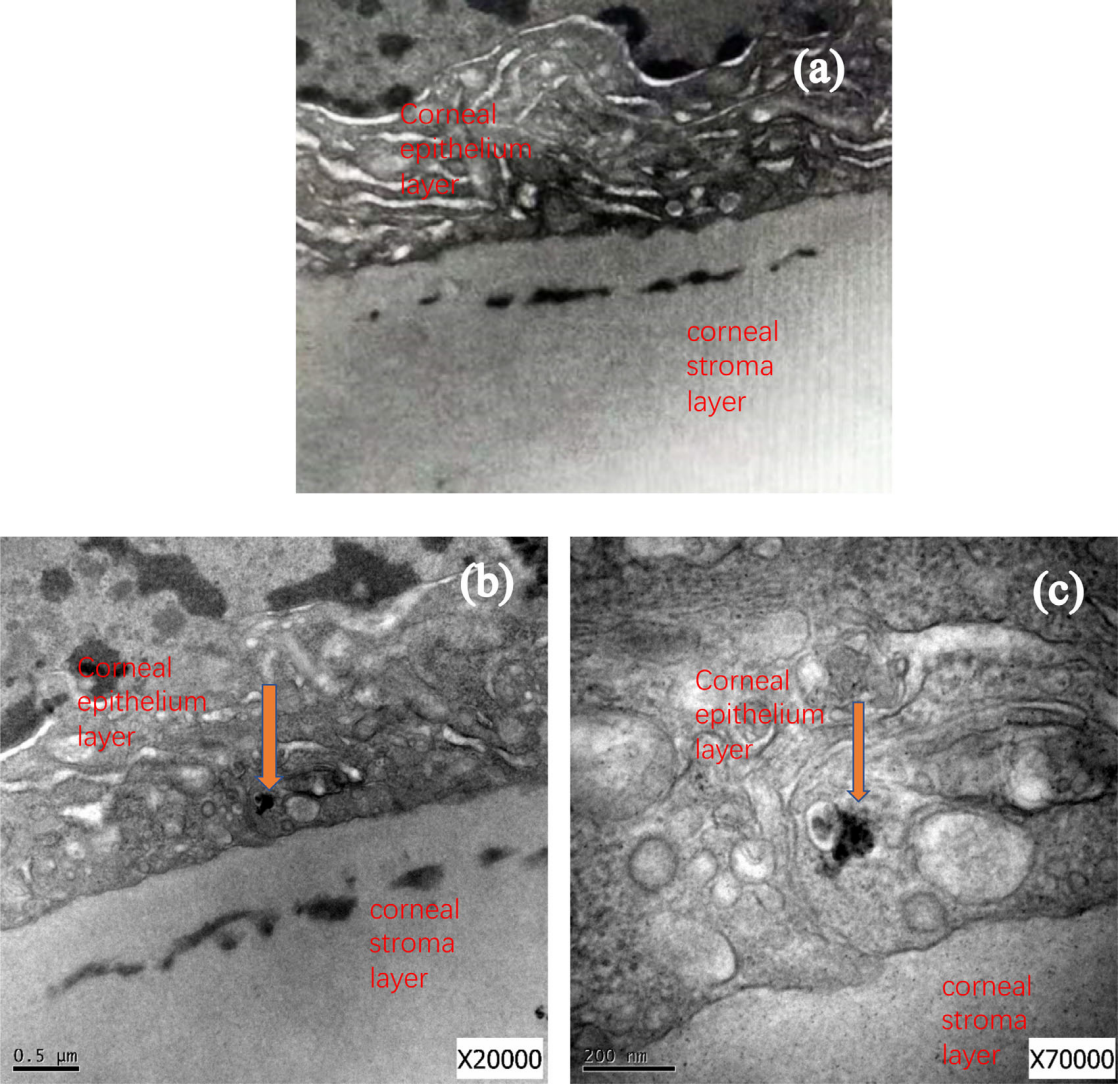
TEM images of the (**A**) control and (**B, C**) corneas treated with carbon nanomaterials (group A).

Figure 5 clearly shows that treated corneas exhibit deep black aggregates at the bottom of the epithelial, most probably due to the presence of carbon nanostructures (see red arrows). As expected, these aggregates are not present in the control group.

In order to improve the observation of the carbon nanostructures, fluorescent-labeled experiments were performed using a IX81 Japan Olympus microscope. To this end, carbon nanostructures were labeled with a fluorescent solution for 30 minutes under sonication before incorporation into the cornea. As shown in Supplementary Figure S4, we first mark at the center of the cornea 3 × 3 mm (purple square area), and then use a 30 G needle pierce to “cross” holes (yellow) in the 4 corners of the square, deep about 100 µm, infiltration for 1 minute, rinse with PBS solution, remove the 3 mm × 3 mm area in the center of the pupil, and puncture the cornea in the reverse direction from the endothelium with a puncture knife. There are 5 pieces in total, each piece is about 20 µm depth. Samples of fluorescence and its morphology were analyzed under a fluorescence microscope at different magnifications.

Supplementary Figure S4 clearly shows that the distribution of the carbon nanostructures varies from layer to layer. From the first (a/A) to the third film (c/C), about 60 µm before the corneal stroma, microscopy images anticipate a large concentration of nanostructures in the upper layer (a/A) with a distribution concentrated mainly along the pinholes, although associated with a high dispersion (green spots can be observed over the whole film under high magnification). Deeper films (b/B and c/C) show less agglomeration of carbon nanostructures, although highly dispersed carbon nanostructures can be appreciated in the two pieces. Films deeper than 60 µm were also evaluated but no fluorescent signal was detected, thus anticipating the absence of carbon nanostructures in the inner corneal layers.

## Discussion

This study confirms that nanotechnology can be considered as a proper alternative to the cross-linking to improve the biomechanical properties of the cornea. Although carbon nanostructures (single-walled carbon nanotubes and graphene) are considered a priori hydrophobic in nature, a proper dispersion in the physiological solution can be achieved using a sonication treatment. Once a homogenous suspension is obtained, carbon nanostructures can be incorporated in the cornea. In order to promote penetration, the epithelium has to be removed and additional punctures performed. Characterization results show that carbon penetration is a diffusion-controlled process and depends on the size and shape of the nanostructure and the residence time. Apparently, carbon nanotubes exhibit a faster diffusion (and associated penetration) compared to graphene, these values being improved several days after incorporation.

The inflation test constitutes the most reliable approach to simulate the stress suffered by the corneas in the specific case of keratoconus. Application of this test to the corneas modified with the cross-linking technology (UV-A + riboflavin) show that the improvement of the tangent elastic modulus is as high as 198% at 0.001 MPa and 172% at 0.01 MPa. These excellent numbers confirm previous results described in the literature for the cross-linking technology.^1^ The large improvement in the strength can be associated with the formation of strong covalent bonds in between the collagen fibers in the corneal stroma after UV-A irradiation. Similar tests performed after incorporation of the carbon nanostructures show that the biomechanical properties are also highly improved, with values around 155% and 141% improvement in the tangent elastic modulus at 0.001 and 0.01 MPa, respectively. The beneficial effect of the nanostructures must be associated to their incorporation in the interfibrillar space and the establishment of strong carbon-collagen interactions in the final composite. Although we cannot reach the excellent properties of the actual cross-linking technology, our results show for the first time in the literature that incorporation of carbon nanomaterials in corneal stroma can reduce significantly the deformation upon an inflation pressure (e.g. after an internal ocular pressure associated with diseases like keratoconus), with the associated improvement in the mechanical properties. The improvement in apical displacement and tangent modulus after incorporation of carbon nanostructures are in correlation with previous studies described in the literature where a beneficial effect of carbon nanostructures in the mechanical properties of composite scaffold polymers was observed.^20^^–^^22^ However, to our knowledge, this is the first time that the improved mechanical properties are observed in vivo using animal models, instead of artificial platforms. Taking into account the long-term and short-term complications documented for the cross-linking technology, in addition to the patient inconvenience due to the use of UV-A irradiation, incorporation of carbon nanostructures in the cornea can be proposed as a promising and novel alternative to treat ocular disorders, such as keratoconus.

## Supplementary Material

Supplement 1
