# Supplementary material for: Carbon Nanostructures for Ocular Tissue Reinforcement
Source: Transl Vis Sci Technol. 2022 Sep 1;11(9):1. doi: 10.1167/tvst.11.9.1 (PMC9440608; doi:10.1167/tvst.11.9.1)
Supplement: Supplement 1 [file tvst-11-9-1_s001.docx]

Carbon Nanostructures for Ocular Tissue Reinforcement

Joaquin Silvestre-Albero, Shihao Chen, Zheng Zheng, Alfredo Vega, Tong Chen, Francisco Rodríguez-Reinoso, Pin Zhu, Shuang Zeng, Yaru Zheng, Fangju Bao, Yong Liu,* and Jorge L. Alió*

To the memory of Prof. Rodríguez-Reinoso

Prof. Dr. J. Silvestre-Albero, Prof. Dr. F. Rodríguez-Reinoso

Laboratorio de Materiales Avanzados, Departamento de Química Inorgánica-IUMA, Universidad de Alicante, Spain

Email: joaquin.silvestre@ua.es

Dr. A. Vega, Dr. L. Bataille, Prof. Dr. J.L. Alió
Research and Development Department, VISSUM Corp., Alicante, Spain

E-mail: jlalio@vissum.com

T. Chen, P. Zhu, S. Zeng, Z. Zheng, Y. Zheng, F. Bao, Prof. Dr. Y. Liu, Prof. Dr. S. Chem

Eye Hospital, Wenzhou Medical University, Wenzhou, China

E-mail: yongliu@wmu.edu.cn

SUPPORTING INFORMATION

|  | **Figure Caption** | **Page** |
| --- | --- | --- |
| **Figure S1** | Experimental device used for the biomechanical inflation testing and specific detail of the fixing device. | 2 |
| **Figure S2** | Transmission electron microscopy images (TEM) of (a) SWCNTs and (b) graphene. | 2 |
| **Figure S3** | Raman spectra of (a) SWCNTs and (b) graphene. | 3 |
| **Figure S4** | Fluorescence microscope images of the first piece (a/A), the second piece (b/B) and the third piece (c/C) corneas treated with labeled fluorescent carbon nanomaterials. | 4 |


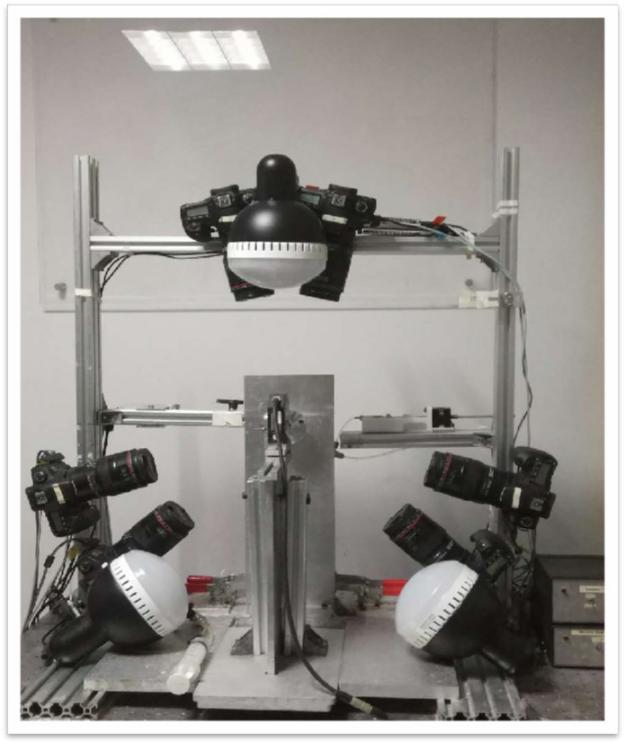

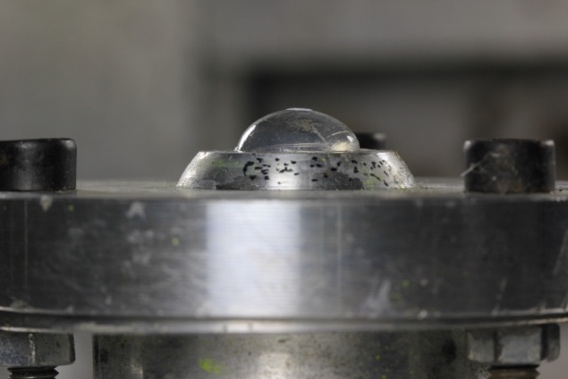


**Figure S1.** Experimental device used for the biomechanical inflation testing and specific detail of the fixing device.

| *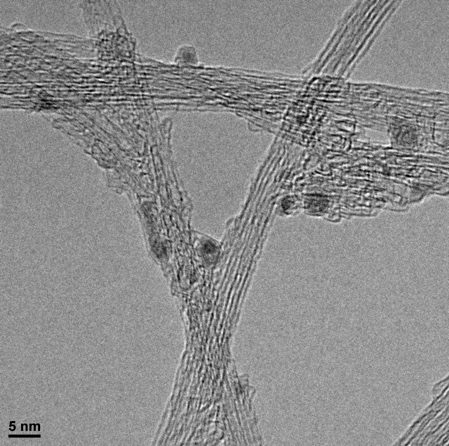*  (a) | *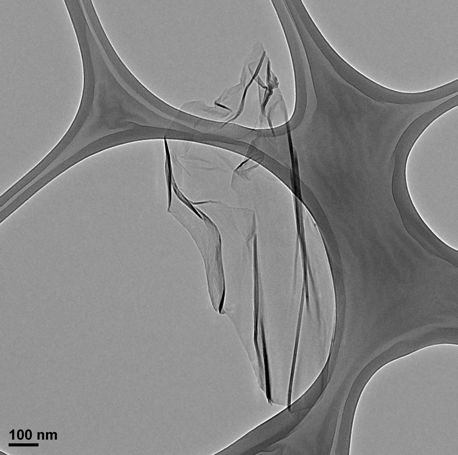*  (b) |
| --- | --- |

**Figure S2.** Transmission electron microscopy images (TEM) of (a) SWCNTs and (b) graphene.

RBM band

D band

G band

G´ band

D band

G band

2D band

**Figure S3.** Raman spectra of (a) SWCNTs and (b) graphene.

| 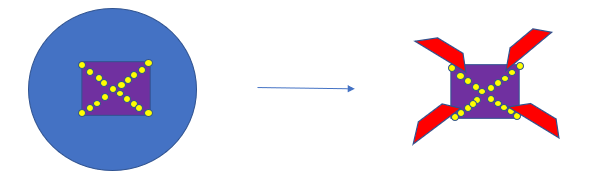 |
| --- |
| 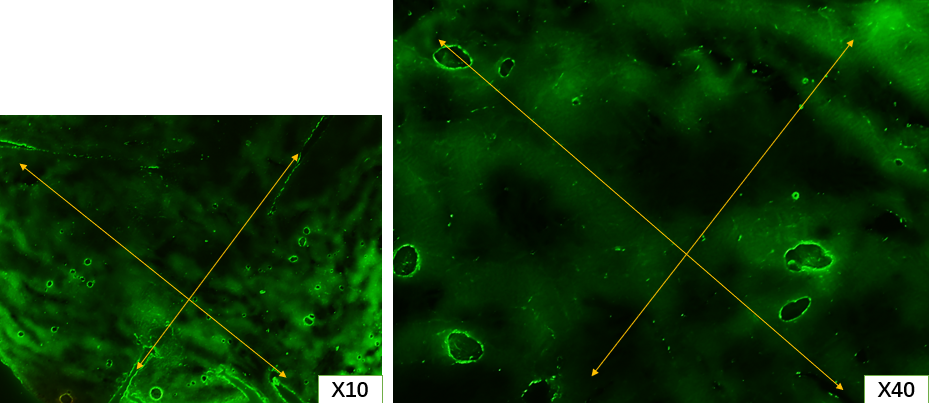  (A)  (B)  (a) |
| 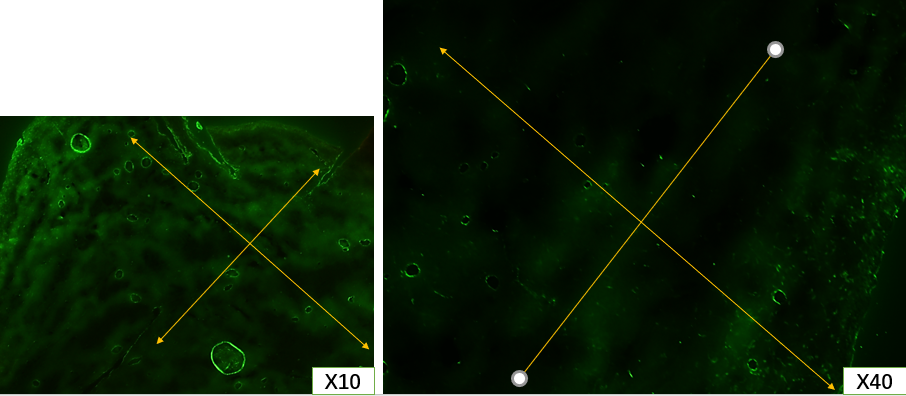  (b) |
| 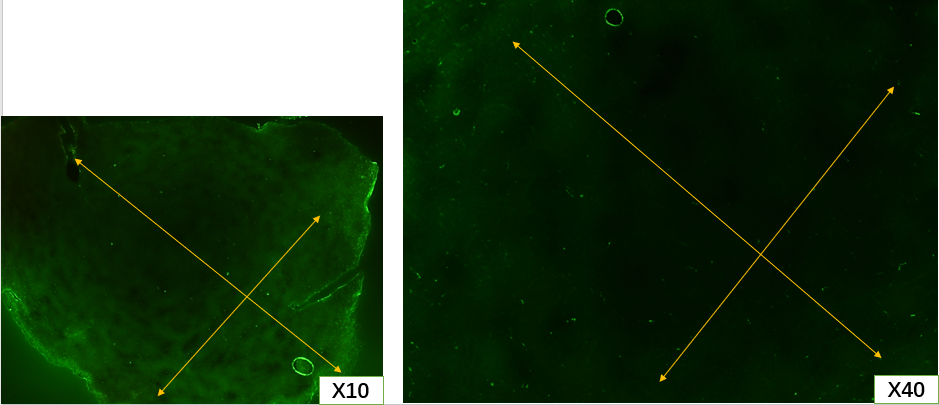  (C)  (c) |

**Figure S4**. Fluorescence microscope images of the first piece (a/A), the second piece (b/B) and the third piece (c/C) corneas treated with labeled fluorescent carbon nanomaterials.
